# Validation of an enzyme-linked immunosorbent assay developed for measuring cortisol concentration in human saliva and serum for its applicability to analyze cortisol in pig saliva

**DOI:** 10.1186/s13028-014-0055-1

**Published:** 2014-09-06

**Authors:** Ola Thomsson, Bodil Ström-Holst, Ylva Sjunnesson, Ann-Sofi Bergqvist

**Affiliations:** Division of Reproduction, Department of Clinical Sciences, Swedish University of Agricultural Sciences, SLU, Box 7054, SE-750 07 Uppsala, Sweden; Division of Diagnostics and Large Animal Clinical Sciences, Department of Clinical Sciences, Swedish University of Agricultural Sciences, SLU, Box 7054, SE-750 07 Uppsala, Sweden

**Keywords:** Stress, Porcine, Sow, Group housing, Multiparous, Primiparous

## Abstract

**Background:**

The purpose of this study was to validate a commercially available enzyme-linked immunosorbent assay (ELISA) developed for measuring free cortisol in human saliva and total cortisol concentration in diluted human serum, for its applicability in measuring cortisol concentration in pig saliva. Collection of saliva is less stressful than e.g. blood sampling, and is a non-invasive method.

**Findings:**

Saliva was collected by allowing sows to chew on cotton swabs held by forceps. Thereafter, the swabs were centrifuged to retrieve the saliva. The ELISA was performed according to instructions provided by the manufacturer. To validate the ELISA, determination of the intra-assay coefficient of variation (CV), inter-assay CV, recovery, linearity and parallelism was performed. The intra-assay CV was below 10% and inter-assay CV below 15% for samples of high, medium and low cortisol concentrations. The mean recovery was 117% and the linearity and parallelism showed an r^2^-value of 0.994 and 0.993, respectively. For biological assessment of induced social stress, two saliva samples were collected in the morning from 6 primiparous and 21 multiparous sows. One sample was collected when the sows were individually housed in a farrowing pen and a second sample was collected when the sows were group housed. The primiparous sows had a significant higher cortisol concentration compared to the multiparous sows when group housed.

**Conclusion:**

The results obtained in this validation study indicate that the ELISA is suitable for measuring cortisol concentration in porcine saliva.

## Findings

Cortisol is a stress hormone produced by the adrenal glands and is commonly measured to assess stress in a variety of animals, including pigs. Cortisol can be measured in plasma or serum [1,2]. However, blood sampling requires either restraint of the pig during sampling, thus being exposed to stress, or surgical insertion of a catheter, which is difficult in large-scale studies or studies conducted in less controlled housing environments [3]. Sampling of saliva, urine and faeces is non-invasive compared to blood sampling. Cortisol metabolites can be measured in urine, but to obtain accurate results urine concentration must be taken into account. Furthermore, urine sampling can be time-consuming as an observer must be present to collect urine when the sow urinates [4]. On the other hand, faeces is easy to collect. A drawback can be that peaks of cortisol are difficult to detect, since the obtained concentration will be a reflection of the cortisol production over time [5]. Saliva is easy to collect and salivary cortisol reflects the biologically active, unbound cortisol in plasma [6,7]. Therefore, collection of saliva is a suitable sampling technique in less controlled environments such as when pigs are mixed and group housed.

Group-housing of sows has been reported to be stressful due the establishment of a new order of rank, usually by more or less violent encounters [8]. It has also been reported that mixing of pigs has an effect on cortisol levels as it is considered a result of social stress [9–11]. Further young sows experience more social stress when housed together with older sows and young sows have a higher cortisol level compared to the older sows [12]. An increase in cortisol levels could also be linked to a reduced on-farm animal welfare [13]. In Swedish organic and conventional piglet production, mixing and group-housing of sows has been practiced since the 1980s and it is also becoming more common within the European Union. Thus, to conduct stress research easily on mixing and group-housing of sows, a combination of saliva collection and a simple analytic method would be useful.

There are several methods for analyzing cortisol in saliva [1,2,14,15], the majority of which being immunoassays. Enzyme-linked immunosorbent assays (ELISA) are widely used in laboratories today. To the authors knowledge there is no commercially available ELISA that has been validated for quantifying cortisol in pig saliva. The aim of the present study was to validate a commercially available ELISA developed for measuring free cortisol in human saliva and total cortisol concentration in diluted human serum, for its applicability in measuring cortisol concentration in pig saliva.

Validation was made both methodologically and biologically. Saliva from five multiparous and one primiparous sow were used for the methodological validation. One multiparous sow was only used for investigation of linearity. The primiparous sow and two multiparous sows were used for recovery studies. One of these multiparous sows, together with two other sows, was also used for calculations of CV, and for the biological validation. A total of 27 sows, 21 multiparous and six primiparous sows, were used for the biological validation. All sows included in this study were healthy pure-bred Yorkshire sows. The study was approved by the Uppsala Animal Ethical Committee (C154/11).

Mixing and group housing of 27 sows was used as a model for social stress as it is known to induce stress [9–11]. One morning saliva sample was collected when the sows were still housed in individual farrowing pens and another morning sample was collected when the sows had been group housed for 12 h. The group size varied between 5 and 8 sows.

Saliva was collected using a Sarstedt Salivette® for saliva collection (ref. 51.1534, Sarstedt, Nümbrecht, Germany). The cotton swab was held with forceps and the sow was allowed to chew on the cotton swab until it was soaked. The swab was centrifuged at 2400 × *g* in room temperature, for 2 min using a Hettich Centrifuge EBA 20 (Andreas Hettich Group, Ltd., Tuttlingen, Germany), within 30 minutes after collection. The recovered saliva was aliqouted and stored at −20°C until analyzed.

A competitive ELISA (Cortisol ELISA, IBL International, Hamburg, Germany) developed for quantitative analysis of free cortisol in human saliva and the total cortisol concentration in diluted human serum was used. According to the manufacturer the limit of detection was 0.005 μg/dl. The assay was performed according to the instructions provided by the manufacturer. The saliva samples were thawed and centrifuged at 3000 × *g* at room temperature for 10 min using Hereaus Fresco 17 centrifuge (ThermoScientific, West Sussex, United Kingdom). The plate was shaken for 2 h using an orbital shaker (Flow Laboratories DSG Titertek, Pforzheim, Germany). For the washing procedure an automatic plate washer Hydroflex Tecan (Tecan Group, Ltd., Männerdorf, Switzerland) was utilized. Optical density was measured by a Multiscan EX (Thermo Labsystems, Vantaa, Finland). The results were calculated using four-parameter-logistic as recommended by the manufacturer.

To calculate recovery, saliva samples from sow A, B and C, 80 μl were mixed with accompanying standards in ratio of 1:1 and pipetted into wells in triplicates. Undiluted samples were analysed to determine the cortisol concentration of the three samples. The concentration was rounded to two decimal places. Recovery was calculated using the formula: (detected concentration/expected concentration) *100. Expected concentration was calculated using the formula: (concentration of undiluted sample + concentration of the added standard) *0.5.

Samples of high, medium and low concentration in two replicates were used to calculated intra-assay and inter-assay coefficients of variation according to Jaedicke *et al.* [16]. Acceptable range was determined for intra-assay CV to 10% and inter-assay CV to 15% [17].

To determine linearity and parallelism, a sample of known high concentration of cortisol and the high concentration accompanying standard were diluted 1:2, 1:4 1:8, 1:16, 1:32 with zero standard and pipetted in to wells in triplicates.

The differences in cortisol concentrations between primiparous and multiparous sows in samples collected before and after group housing were compared. A Shapiro-Wilk test showed that the cortisol concentrations were not normally distributed and the analysis was therefore conducted using the Wilcoxon rank-sum test. A *P*-value < 0.05 was considered significant.

**Figure 1:**
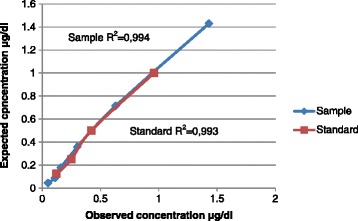
**Investigation of linearity and parallelism through a dilution of a sample with high concentration of cortisol and dilution of a high concentration of cortisol accompanying standard.**

The recovery is presented in Table 1. The mean recovery for sows A, B and C were 110%, 89% and 154%, respectively. The overall recovery was 118%. The results of the linearity and parallelism are presented in Figure 1, whereas Table 2 shows the intra-assay and inter-assay CVs. Intra-assay CV ranged from 0.9% to 4.8% and the inter-assay CV ranged from 5.9% to 14.8%.

**Table 1:** **Recovery rate in three samples from 3 sows (A, B and C) with different cortisol concentration mixed with accompanying standards**

**Added concentration** ^**1**^ **μg/dl**	**Sow A (0.58 μg/dl)** ^**2**^			**Sow B (0.42 μg/dl)** ^**2**^			**Sow C (0.54 μg/dl)** ^**2**^		
	**Expected conc.**	**Detected conc.**	**Recovery %**	**Expected conc.**	**Detected conc.**	**Recovery %**	**Expected conc.**	**Detected conc.**	**Recovery %**
0.03	0.31	0.37	119	0.23	0.21	91	0.29	0.42	145
0.06	0.32	0.34	106	0.24	0.19	79	0.30	0.48	160
0.20	0.39	0.42	108	0.31	0.24	77	0.56	0.56	151
0.60	0.59	0.57	97	0.51	0.49	96	0.57	0.91	160
1.50	1.04	1.31	126	0.96	0.90	94	1.02	1.78	175
4.00	2.29	2.38	104	2.21	2.08	94	2.27	3.00	132

^1^Concentration added in 1:1 ratio.

^2^The undiluted cortisol concentration of the sample.

**Table 2:** **Intra-assay and inter-assay coefficients of variation of samples with high, medium or low cortisol concentrations**

**Sample concentration**	**Intra-assay CV**			**Inter-assay CV**		
	**Mean μg/dl**	**SD**	**CV %**	**Mean μg/dl**	**SD**	**CV %**
High	3.23	0.16	5.0	3.42	0.20	5.9
Medium	2.25	0.04	1.8	1.96	0.29	14.8
Low	0.55	0.01	1.8	0.48	0.06	13.0

The concentrations of cortisol did not differ significantly (*P* = 0.81) in morning samples collected from single housed multiparous (median 0.47 μg/dl, range 0.14-2.53 μg/dl) compared to single housed primiparous sows (median 0.66 μg/dl, range 0.10 - 1.17 μg/dl). However the concentration of cortisol did differ significantly (*P* = 0.03) in morning samples collected during group housing between multiparous and primiparous sows, with a higher concentrations for the primiparous sows. The median for the primiparous sows was 0.44 μg/dl (range 0.50 -1.23 μg/dl) and for multiparous sows the median was 0.31 μg/dl (0.08- 0.60 μg/dl) for the samples after group housing.

In this study the overall recovery reported was lower than in other validation studies of cortisol measured in saliva with different immunoassays [14,18]. However it was still within acceptable range of 80%-120% [16]. The higher overall recovery was mainly due to the over-recovery of sow C. The intra-assay CV was below 10% and the inter-assay CV was below 15% for all sample concentrations which is acceptable [17]. The r^2^- value was close to unity for both the diluted sample and the diluted high standard and, together with an acceptable recovery, indicates a high accuracy [14]. Together these results indicate that the ELISA is suitable for analyzing cortisol concentrations in pig saliva.

In the biological validation of the present study the cortisol concentrations differed significantly between multiparous sows and primiparous sows when group housed. The same difference with higher cortisol concentrations for primiparous sows compared to multiparous sows at group housing has previously been reported as a result of socially induced stress [12,19]. The result obtained from the mixing of multiparous sows and primiparous sows in this study is supported by results in previous studies. The cortisol levels also appeared to be lower in group housed compared to individually housed sows regardless of parity, corresponding to previous studies [20,21].

In conclusion the results of this validation imply that the cortisol ELISA provided by IBL International is suited for analyzing cortisol concentrations in pig saliva.
